# Health in Our Hands: diabetes and substance use education through a new genomic framework for schools and communities

**DOI:** 10.1007/s12687-022-00631-x

**Published:** 2023-01-16

**Authors:** Stephen M. Modell, Irene S. Bayer, Sharon L. R. Kardia, Consuelo J. Morales, Idit Adler, Ella Greene-Moton

**Affiliations:** 1 Center for Public Health and Community Genomics, University of Michigan School of Public Health, M5049 SPH II, 1415 Washington Hts., Ann Arbor, MI 48109-2029 USA; 2 CREATE for STEM Institute, Michigan State University, East Lansing, MI USA; 3 Department of Epidemiology, University of Michigan School of Public Health, Ann Arbor, MI USA; 4 Tel Aviv University Constantiner School of Education, Tel Aviv, Israel; 5 Community Based Organization Partners (CBOP), Flint, MI USA

**Keywords:** Program evaluation, Mixed methods research, Problem-based learning, Schools, Science education, Health education, Gene-environment interaction, Diabetes, Substance use disorder

## Abstract

From May 2014 through June 2019, educational, health, and academic partners under an NIH Science Education Partnership Award (SEPA) engaged 1271 6th through 8th grade students and their families in the “A New Genomic Framework for Schools and Communities” program. Evaluation addressed the effectiveness of the Health in Our Hands genomics curriculum, which employed Next Generation Science Standards and community action research projects to target two common, complex conditions—type 2 diabetes and substance use disorder (SUD)—in the underserved cities of Flint and Detroit, MI, USA. Curriculum outcomes were measured with classroom surveys, presentation event questionnaires, and adult interviews using mixed qualitative/quantitative (SPSS V. 25.0) methods involving generalized linear mixed modeling-based ANOVA. The diabetes unit enactment registered a 12% pre- /post-gain among students in perceived learning about genes and the environment. Both diabetes and SUD units showed statistically significant gains in perceived learning about health and health conditions and the importance of what students were learning to everyday life. A total of 73.4% of fall 2018 SUD event participants indicated increased awareness of educational and career choices in science. Moderate gains were noted during the diabetes curriculum in students sharing what they learned with friends and family. 9/11 parents and 5/9 community members attending the student presentation event had discussed diabetes with a student. Linked formal classroom and informal community-connected approaches can successfully be used to teach genomics and promote project-based learning in students, family, and community members. Further efforts are needed to effectively engage families.

## Introduction

The coronavirus pandemic clearly illustrates the need for scientific literacy about disease risk and prevention. However, developing students’ understanding of the ideas involved—what occurs at the microscopic and molecular levels and the evolving interplay between an organism’s genetic makeup and the environment—poses challenges to science educators. Advances in our understanding of how students learn science, synthesized in the 2011 National Research Council of the National Academy of Sciences report *A Framework for K–12 Science Education*, help address these challenges through a vision for the scope and coherence of science and engineering education (National Research Council 2012). Teaching should be 3-dimensional (Krajcik et al. 2014), addressing (1) disciplinary core ideas focused on a limited area important to students’ understanding of science, such as gene-environment interactions; (2) cross-cutting concepts that span multiple scientific domains, such as patterns and cause and effect; and (3) scientific practices that scientists employ to figure out phenomena, including asking questions, planning and carrying out investigations, and modeling. The term “scientific practices” is used instead of “inquiry” since the latter have been interpreted in many different ways by the science and health education communities.

### Next Generation Science Standards

In 2013, the Next Generation Science Standards (NGSS), based on the *Framework*, were released for state use to set expectations for what students should know and be able to do at each grade level or grade band (NGSS Lead States 2013). Structure and content in the new scientific framework adhere to several major educational guiding principles: children’s capacity to learn science, the development of meaningful learning over time, the linkage of science education to students’ interests and experiences, and equity promotion (National Research Council 2012). The NGSS are laid out as clearly defined “performance expectations” (e.g., MS-LS1-5: construct a scientific explanation based on evidence for how environmental and genetic factors influence the growth of organisms; MS-LS1-8: gather and synthesize information that sensory receptors respond to stimuli by sending messages to the brain for immediate behavior or storage as memories) (Krajcik and Czerniak 2018; Michigan Dept. of Education 2015). To date, 20 states and the District of Columbia have adopted the NGSS, with Michigan being a lead state in the process, and another 24 states have adapted their science standards based on the *Framework* recommendations (National Science Teaching Association 2022; Michigan Department of Education 2015).

### Project-based learning

Project-based learning (PBL) is an approach to science teaching that can be used to actualize the vision of the *Framework* in the classroom (Krajcik and Shin 2014). Based on constructivism (the learner’s active involvement as critical for constructing new personal knowledge) (Bachtold 2013) and situated learning (knowledge at its best lives and grows in context) (Sadler 2009; Ackermann 2001), PBL is driven by students’ questions about a phenomenon they can observe or experience. Students investigate collaboratively, using technology such as online simulations to scaffold their learning and creating artifacts such as models and explanations that help describe and explain their understanding. Tal and colleagues suggest that using engaging curriculum materials including computers along with standards-based science curriculum materials can help minority students within urban school districts short of educational resources to narrow achievement gaps (Tal et al. 2006). The science classroom can then connect with resources and experts in students’ communities, i.e., to community assets, through activities that strengthen student learning while advancing scientific literacy among family and community members (Krajcik et al. 2022; Nguyen and Siegel 2015).

In the study of genetics, students are often led through a conceptual progression beginning with an appreciation of the genetic information within organisms and ending with the influence of environmental factors on gene expression (Duncan et al. 2009). The learning rarely expands, however, into the students’ own lives or family health circumstances and their community environment (Nation et al. 2003). Furthermore, the examples used are often constrained to simple Mendelian conditions like sickle cell disease and albinism (Hurle et al. 2013). Dougherty and colleagues have argued that public- and school-based genomic literacy efforts need to incorporate common complex conditions people experience everyday—“cardiovascular disease, diabetes, and mental illness” (Dougherty et al. 2014, 2011). This paper describes an educational research study prioritizing NGSS and PBL principles for middle school science and assesses the program’s merits in teaching genetics to middle school students and their communities in two real-world contexts—type 2 diabetes and substance use disorder (SUD).

## Materials and methods

### Study design

From October 2014 through May 2019, researchers from the Michigan State University CREATE for STEM Institute and the University of Michigan School of Public Health, funded by a US National Institutes of Health Science Education Partnership Award (SEPA), worked with teachers to engage middle school students from two underserved Michigan cities in the “A New Genomic Framework for Schools and Communities” genetics educational program. Program goals were to further knowledge for students and adults about gene-environment interactions and their impact on human health.

**Fig. 1:**
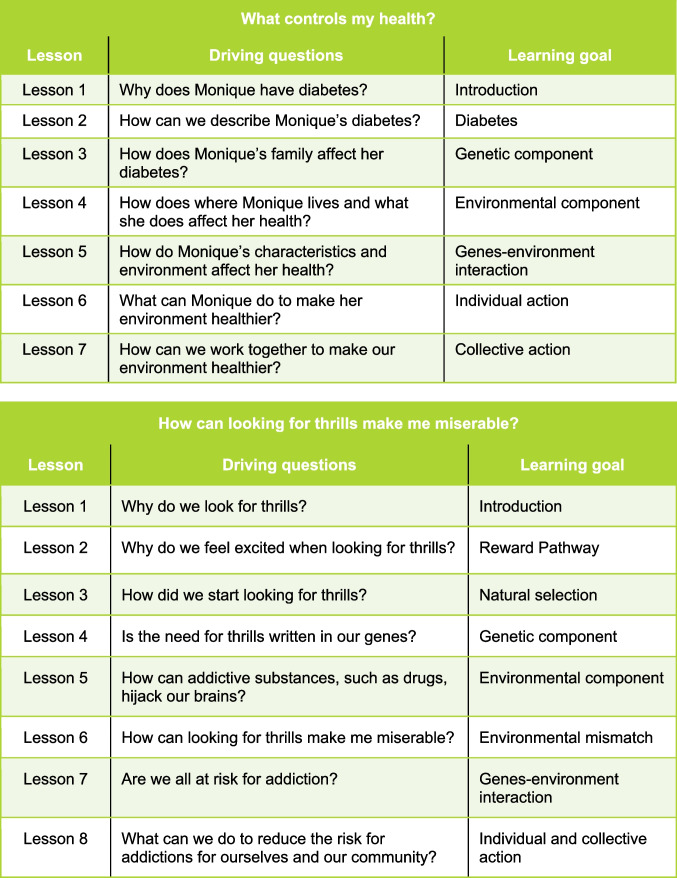
Health in Our Hands middle school units, 2017

The program followed a pre-post design, using a mixed methods approach to develop and study two middle school science curricular units called Health in Our Hands (HiOH) (Adler et al. 2020, 2017; Axinn and Pearce 2006; Campbell and Stanley 1966). The first unit, taught in 6th grade, focused on type 2 diabetes; the second, taught in 7th and 8th grades, focused on SUD, embodying different forms of addictive behavior. Learning materials were designed according to NGSS and utilized a PBL approach that connected with the community. School district science teachers and administrators, community-based and health organizations, and university education, science, and public health researchers collaborated in the design, enactment, and evaluation of the curricular materials outlined in Fig. 1.

In Unit 1, *What controls my health?,* students meet Monique, a girl diagnosed with type 2 diabetes, by video. Students explore the biology of diabetes and investigate how lifestyle options for healthy foods and exercise can help prevent the condition (CDC 2021). Unit 2 *How can looking for thrills make me miserable?* starts with a video of teens’ testimonials about addiction to vaping (e-cigarettes). Students investigate the brain’s reward system from an evolutionary perspective and examine its role in addictive behavior. To evaluate perceived gains, student and teacher surveys were collected for both units at the beginning and end of each 10–12 week semester.

For each unit, project researchers provided teachers with a detailed teacher’s guide and extensive professional learning starting with three full-day sessions of professional learning and providing continuing support throughout the semester. Student in-class activities included (1) a sand rat simulation to model diabetes under different food conditions and genotypes (Lee et al. 2018); (2) simulated blood plasma samples using “Science Take-Out” hands-on learning kits to test for the presence of type 1 or type 2 diabetes (Alcena-Stiner and Markowitz 2020); and (3) for the SUD unit, a lab from the “Science Take-Out” hands-on learning kits to examine how a new drug, “Floratryp,” can hijack the brain’s reward pathway and lead to addictive behavior in mice and humans (Alcena-Stiner and Markowitz 2020).

At the end of both units, students conducted a “community action research project” (CAP) to figure out, “How can we work together to make our community healthier?” CAPs innately carry an element of creative openness about how the project pieces progress and what students will figure out that instills curiosity in students (Nguyen and Siegel 2015). That quality plus their relevance to the community draws students in. In their CAP, students collaborated on formulating a research question, identified relevant components of the environment, developed instruments for measuring change, and conducted their projects within the community. CAPs included (1) exploring whether raising students’ awareness of the amount of sugar they eat affects their food choices; (2) holding a smoothie contest to study strategies for influencing healthy choices; and (3) investigating (through surveys of family members, friends, and other students) how the use of social media (cell phones, video games) affects feelings of well-being. The CAP topics are not only engaging to the students but can be highly relevant to their lived experiences. Multidisciplinary genome investigator Dr. Gil Omenn succinctly explains: “Drugs of abuse create signals in the brain that indicate falsely the arrival of a fitness benefit such that drug seeking behavior displaces adaptive behaviors. Video game playing and snacks high in fat, salt, and sugar [are] described in similar terms” (Omenn 2010). Mentors with expertise in relevant areas (e.g., nutrition, urban planning) and drawn from within the community and universities were interviewed by students and, in some cases, visited the classroom multiple times and provided help and support to students’ CAPs. Finally, students from across schools and districts then came together in a closing event, the Health Summit, with peers, family members, and community partners to present their findings and recommendations for community action. Participant event questionnaires and adult interviews were collected on these occasions. The projects and student presentations were used to assess to what extent a middle school curriculum that focuses on critical community health concerns can convey the core idea of gene-environment interactions affecting health.

### Recruitment and data collection

Schools were selected based on previous collaborations in a SEPA-funded project with high schools in Southeast Michigan that concluded in 2012 (Alozie et al. 2010). Our community partners also suggested contacts, both teachers and schools, that we might want to approach. We spoke with teachers throughout the county and state-wide to inform them about the curriculum and discuss whether they were interested in teaching it in their classrooms. The current study enrolled 1271 6th through 8th grade students from 2016 to 2018 from Flint and Detroit, both in HRSA-identified medically underserved areas (Department of Health and Human Services, Health Resources and Services Administration 2020). Flint students participated in pilot and field testing and final enactment of both educational units; Detroit students in pilot and early field testing of the diabetes unit. All procedures and forms were approved by the city administrative offices of curriculum and instruction, a community ethics review board, and the two universities’ health and behavioral sciences institutional review boards (IRBs).

### Measures

Demographic information included student grade, teacher, and class hour. Student surveys were affixed with school name and semester. Collection of data on student sex (male or female) began in spring 2017, a year after the diabetes unit pilot data collection started. Program evaluation was based on quantitative pre- /post-surveys administered to students and teachers, mixed quantitative–qualitative event questionnaires, and adult interviews (Table 1). The former contained seven question items with 3-point Likert scales for student understandability (Mellor and Moore 2014). The team made the pragmatic decision of adopting a 3-point scale for the class-related surveys following the observation during pilot testing that students were not using the full range of point categories along a 5-point scale for this instrument. Event questionnaires used closed- and open-ended questions to register personal event impact and satisfaction, while adult questionnaires assessed program awareness, student-adult interaction, and project involvement. The adult interviews contained a prearranged list of open-ended questions, divided into questions specifically for parents and those for all adult participants. Instrument wording was initially checked with project board members (educational administrators, an evolutionary biologist, and community leaders) and went through review iterations by a community activities and evaluation committee with each successive semester.

**Table 1:** Survey and questionnaire summary

Year/unit	Enactment/location	#studnt	#teachrs	#studnt pre/post surveys	#teacher pre/post surveys	#student event questionnaires	#teacher event questionnaires	#adult event questionnaires	#parent interviews	# all adult interviews
2016/diabetes	Pilot Detroit & Flint	162	3	90/120	1/2	115	4	61	2	8
Spring 2017/diabetes	Field test Detroit & Flint	372	9	279/249	7/0	59	0	30	5	9
Fall 2017/diabetes	Enactment Flint & Clio	309	8	251/234	8/8	197	7	20	0	10
Spring 2018/substance use disorder (SUD)	Pilot Flint & Flint Township	139	1	131/119	1/1	111	1	14	6	13
Fall 2018/SUD	Field test/enactment Flint & Flint Township	289	3	249/220	3/1	182	2	12	5	6

### Analysis

Pre- /post-comparisons of student surveys used during 6th grade (spring and fall 2017 semesters) and 7th–8th grades (spring and fall 2018 semesters) field testing and final enactment were performed using IBM SPSS V. 25.0. Frequencies for the various question items were assessed by year and season. SPSS generalized linear mixed modeling was used to generate ANOVA tables listing the significance level (post- to pre-comparison) for each of the student survey question items. Time (*T* = 0; *T* = 1) was the principal fixed effect, with sex and class hour (i.e., particular group of students) as covariates. Survey items found to be statistically significant on class hour were examined further for those instructors teaching the curriculum in more than two classes per semester. Teacher experience (number of prior semesters having taught the curriculum) and whether a student had taken the other unit in the previous grade (iteration variable) served as additional covariates when sufficient variation existed to enable their inclusion. Student ID and teacher ID were jointly used as random effects except in two specific instances where teacher ID was used to enable convergence (see Table 3, f.n. 7). Data from teachers who did not return end-of-unit student surveys for a given semester were used in the presentation event analysis, but excluded from the pre- /post-analysis.

Analyses of the participant event questionnaires were conducted by year and season (a proxy for the particular semester), with frequency counts and percentages generated for the various question items. These figures were examined by participant type (e.g., student, parent, school personnel) and overall. Crosstabulations were conducted for select independent variables.

## Results

### Participant characteristics

Both cities in which the schools assessed are located have an individual median income of less than $20,000 and reflect a diverse racial-ethnic composition with a predominance of racial-ethnic minority residents (US Census Bureau 2019a, b). Forty-three percent of the Detroit population and 50.1% of the Flint population are enrolled in Medicaid (Data USA 2022a, b). Overall, 843 (66.3%) students were in 6th grade, 360 students (28.3%) were in 7th grade, and 68 (5.4%) were in 8th grade (Table 2).

**Table 2:** Participant characteristics, 2019

Category	Flint, MI	Detroit, MI
City racial-ethnic composition:*		
African American	54.1%	78.3%
European American	39.1%	14.7%
Latino	4.5%	7.7%
Multiracial	5.1%	1.8%
City individual median income*	$17,086	$18,621
Number of participating schools	10 (1 pilot and field testing; 7 field testing only; 2 enactment only)	1 (pilot testing)
Number of participating teachers	16	2
Number of participating students	1158 (91.1%)	113 (8.9%)
Student grade level composition:		
6th grade	730 (63.0%)	113 (100%)
7th grade	360 (31.1%)	0%
8th grade	68 (5.9%)	0%
Student sex breakdown:**		
Male	557 (50.6%)	––-
Female	543 (49.4%)	––-

^*^US Census Bureau 2019

^**^Sex was collected starting spring 2017

### Pilot study

During the 2016 pilot phase of the project in Detroit and Flint, MI, USA, the evaluation team collected and analyzed classroom surveys for 162 students (113 Detroit; 49 Flint). Student surveys demonstrated a statistically significant increase in the perceived importance of genetic knowledge to everyday life (*P* < 0.012). As indicated by the adult and supplemental student interviews, many students and their parents could relate directly to the diabetes examples used. A student teacher commented: “You can’t see your genes, but now the students are able to see how they form their lifestyles and compare to what they’re learning.” Statistically significant increases were also noted in students’ interest in how the environment can affect what we are, and the perceived usefulness of the curriculum for learning about health and disease. A 28-year-old mother attending the Detroit pilot study closing event remarked that her daughter “learned about diabetes in my father and her grandmother and aunt. You can use that information to know symptoms, causes, and effects, and pass on that information to someone who doesn’t.”

The surveys suggested that overall, however, more effort would be needed in motivating students to share what they were learning with friends and family. The diabetes curriculum pilot phase was particularly helpful in simplifying the language used in the questionnaires and introducing additional steps to promote sharing. The spring 2018 SUD curriculum pilot phase, detailed below, was instrumental in highlighting the need for further effort to make presentation event participants aware of future career choices. A parent suggestion that “[project] judges should talk about their own profession” was subsequently incorporated.

### Diabetes unit enactment findings and comparisons

Student responses to survey items were more positive in the fall 2017 final enactment of the diabetes curriculum than during the previous spring 2017 field testing, both occurring in the Flint area. The percentage of students strongly agreeing that the class is useful for learning about genetics and the environment increased by 10% between the two semesters, each representing a different cohort, while the percentage increased by 11% for the question item concerning usefulness in learning about health and disease (Table 3). The number of teachers strongly agreeing with this latter statement in the fall increased from 5/8 (pre) to 8/8 (post).

**Table 3:** 6th grade student survey—what controls my health?

Question item	Level of agreement	# of students Pre (*N* = 314)^1^ (Spring 2017)^2^	# of students Post (*N* = 286) (Spring 2017)	# of students Pre (*N* = 254) (Fall 2017)	# of students Post (*N* = 236) (Fall 2017)
1. In science class, I learn how genetic knowledge can be important to my everyday life	Very true^3^ Somewhat true	28.8% (80)^4^ 44.6% (124)	56.9% (141)**^5^ 40.7% (101)	36.7% (92) 46.2% (116)	58.1% (136)**^6^ 40.6% (95)
2. I am interested in learning about how our characteristics and the environment can affect my health	Very true Somewhat true	57.0% (159) 36.9% (103)	56.2% (140)**^7^ 38.2% (95)	63.2% (158) 31.6% (79)	65.2% (152) 30.5% (71)
3. Science class is useful for learning about genetics and the environment	Very true Somewhat true	56.5% (156) 39.1% (108)	59.9% (148) 36.0% (89)	57.8% (145) 35.1% (88)	69.5% (162)** 27.9% (65)
4. Science class is useful for learning about health and disease	Very true Somewhat true	53.0% (148) 36.2% (101)	68.7% (171)** 28.1% (70)	66.4% (166) 25.6% (64)	79.3% (184)** 17.7% (41)
5. Information obtained outside the classroom is useful for learning about health and nutrition	Very true Somewhat true	33.5% (93) 51.8% (144)	41.0% (102) 52.6% (131)	41.3% (102) 48.2% (119)	42.9% (99)**^7^ 48.9% (113)
6. Learning about genetics will help me make decisions about my educational choices and career path in the future	Very true Somewhat true	49.5% (138) 38.7% (108)	48.2% (119) 41.7% (103)	58.8% (147) 30.4% (76)	52.6% (122) 38.8% (90)
7. I share what we learn in class about genetics and genetic issues with my friends and family	Very true Somewhat true	21.1% (59) 43.7% (122)	32.7% (81)** 44.0% (109)	31.9% (79) 38.7% (96)	42.7% (99)** 40.1% (93)

^1^Spring 2017: 35 non-respondents (pre), 37 non-respondents (post); Fall 2017: 3 non-respondents (pre), 2 non-respondents (post) on at least 1 question item

^2^Spring 2017 and Fall 2017 represent different school years with different 6th grade students

^3^3-point Likert scale

^4^28.8% (80) means “28.8% of the 6th grade students, or a total of 80 students”

^5^Significance levels: *P* < .05 (*); < .005 (**), based on the *P*-value for time (after vs. before intervention)

^6^Sex a significant covariate, *P* < .005

^7^TchrID, rather than StuId by TchrID, used as the random effects variable to enable convergence in these two instances

Both students and teachers registered pre- /post-gains in a question about student interest in the theme of “how our characteristics and the environment can affect my health.” Likewise, student enthusiasm for the online simulation of the effect of genetics and the environment on sand rats’ health was in general positive. In response to open-ended questions in the student interviews and event questionnaires, comments varied, including interest and desire to further study what they eat; one student wrote on the questionnaire form that he did “not like” what he learned about sand rats. Further probing would have been necessary to understand why the student answered this way.

For both spring and fall periods, the curriculum achieved statistically significant pre- /post-gains in students’ responses to the following statements: (A) “In science class, I learn how genetic knowledge can be important to my everyday life”; (B) “Science class is useful for learning about health and disease”; and (C) “I share what we learn in class about genetics and genetic issues with my friends and family” (*P* < 0.005 for each). Teachers also displayed pre- /post-increases in their responses to both learning usefulness questions, though high pretest scores precluded these results from being statistically significant. During the fall, the number of teachers marking “very true” to the item on the importance to everyday life rose from 2/8 teachers (pre) to 7/8 (post). Over the same semester, 42.7% (99/232) of students post- vs. 31.9% (79/248) of students pre-affirmed that they shared what they learned in class with friends and family. The number of teachers strongly agreeing with the statement on student sharing increased from 3/8 (pre) to 5/8 (post). While the above percentages by themselves could be improved, the change was nonetheless statistically significant (*P* < 0.001). In addition, the fall semester showed statistically significant (*P* < 0.003) Pre- /post-increases in students strongly agreeing the class was useful for learning about genetics and the environment and that information obtained outside the classroom was helpful.

Of 30 spring 2017 adult presentation event respondents, 53% (16) marked “a lot” and 20% (6) marked “some” in terms of their prior awareness of the curriculum and projects. Nine of 11 parents or family members and 5 of 9 community members (school employees, student project volunteers, Food Corps volunteers) indicated having talked with a student about diabetes. A 46-year-old community member at the fall presentation event remarked, “One young lady showed how a grandmother has diabetes. The student suggested food to her grandmother – they would pick better choices, not just pizzas.” A parent at the spring event noted, “My son didn’t discuss the diabetes, but the food part with Monique. That kids can get diabetes. Monique is his age.” Parent interviewees described helping students pick up their supplies, taking their student’s survey as part of their project, and helping transport project boards and I-Pads. Ten of 11 parents or family members affirmed having subsequently discussed making food changes within their family.

### SUD unit enactment findings and comparisons

Pilot testing for the SUD unit occurred in spring 2018, while fall 2018 constituted a combined field testing/final enactment period, both carried out in Flint and Flint Township. Only student responses to survey item 4, perceived curricular usefulness for learning about health and health conditions, registered an uptick from spring (47.5%) to fall 2018 (51.6%) (different cohorts) in the percentage of students who strongly agreed (Table 4). However, both semesters did show statistically significant pre- / post-gains (*P* < 0.05) for three of the question items: (A) perceived curricular usefulness for learning about health and health conditions; (B) perceived usefulness of information obtained from science class activities; and (C) sharing what is learned with friends and family. In addition, during fall 2018, survey item 1, perceived curricular importance to everyday life, showed a statistically significant increase in the percentage of students strongly agreeing, from 22.8% (56/246) pre to 41.3% (90/218) post (*P* < 0.001).

**Table 4:** 7th and 8th grade student survey—how can looking for thrills make me miserable?

Question item	Level of agreement	# of students Pre (*N* = 131)^1^ (Spring 2018)^2^	# of students Post (*N* = 119) (Spring 2018)	# of students Pre (*N* = 249) (Fall 2018)	# of students Post (*N* = 220) (Fall 2018)
1. In science class, I learn how genetic knowledge can be important to my everyday life	Very true^3^ Somewhat true	36.9% (48)^4^ 61.5% (80)	46.2% (55) 53.8% (64)	22.8% (56) 63.4% (156)	41.3% (90)** 96.3% (120)
2. I am interested in learning about how our characteristics and the environment can affect my health	Very true Somewhat true	51.5% (67) 40.8% (53)	47.1% (56) 45.4% (54)	42.2% (105) 47.0% (117)	36.5% (80) 53.4% (117)
3. Science class is useful for learning about genetics and the environment	Very true Somewhat true	69.2% (90) 26.2% (34)	74.4% (87) 23.9% (28)	53.7% (132) 42.7% (105)	58.2% (128) 37.7% (83)
4. Science class is useful for learning about health and health conditions	Very true Somewhat true	27.5% (36) 55.7% (73)	47.5% (56)**^5^ 47.5% (56)	44.6% (111) 45.4% (113)	51.6% (113)*^6^ 43.4% (95)
5. Information obtained from science class activities is useful for learning about addiction	Very true Somewhat true	37.7% (49) 52.3% (68)	50.8% (60)* 45.8% (54)	25.4% (63) 59.3% (147)	37.2% (81)* 87.6% (110)
6. Learning about genetics will help me make decisions about my educational choices and career path in the future	Very true Somewhat true	38.9% (51) 38.9% (51)	38.7% (46) 51.3% (61)	33.5% (83) 45.6% (113)	36.8% (81) 82.7% (101)
7. I share what we learn in class about genetics and genetic issues with my friends and family	Very true Somewhat true	12.2% (16) 38.9% (51)	20.3% (24)* 44.9% (53)	14.6% (36) 37.7% (93)	17.3% (38)* 45.0% (99)

^1^Spring 2018: 1 non-respondent (pre), 2 non-respondents (post); Fall 2017: 3 non-respondents (pre), 3 non-respondents (post) on at least 1 question item

^2^Spring 2018 and Fall 2018 represent different school years with different 7th and 8th grade students

^3^3-point Likert scale

^4^36.9% (48) means “36.9% of the 7th and 8th grade students responding to the statement item, or a total of 48 students”

^5^Significance levels: *P* < .05 (*); < .005 (**) based on the *P*-value for time (after vs. before intervention)

^6^Sex a significant covariate, *P* < .05

More fall 2018 student and adult participants (73.4% or 127/173) showed agreement on presentation event questionnaire item 6, “This event made me aware of educational and career choices in science,” than did spring participants (69.0% or 78/113). Teacher 1’s response reversed from “somewhat disagree” (spring) on this statement about their students’ educational and career awareness to “strongly agree” (fall). One fall student wrote on their event questionnaire, “I liked how they told us how their life was and how they got to where they are at now.” Another commented, “I liked that we got to meet some people who want to make a change in the world.”

In the event questionnaires and interviews, parents and community members focused more on the students’ community projects than the in-class activities. A 26-year-old community member remarked, “I like to hope it will increase their interest in research and science. Learning what they learned helped them grasp what they did in the community.” Likewise, for both curricular units, 5/9 teachers marked “very true” and 4/9 marked “somewhat true” to the teacher survey statement “The community activities outside the classroom are of value to my students.” Furthermore, a fall 2018 teacher who was especially engaged with student projects marked that the closing event, which involved students describing their projects, had motivated him to think about making personal healthy lifestyle decisions. During spring 2018, the majority of adult event participants and half the parents marked “some” as to whether they discussed making healthy lifestyle decisions with a student. Parents discussed topics like drug use and cell phone addiction with their kids and referred to replacement activities such as sports and everyday tasks like mowing the lawn.

Fall 2018 adult presentation event attendees were asked how they first found out about the new science curriculum. Of 9 respondents, 1 heard about the new curriculum from their student; 1 recalled receiving a notifying letter from the school, and 7 indicated they heard about it via an e-mail from the curriculum organizers. Though students are very familiar with Instagram and Facebook, only 2/3 of spring adult attendees and 4/10 of adult attendees for both semesters combined indicated they had visited these program social media pages.

### Subgroup analysis results

In the regression analyses of student survey results, the covariates sex, iteration (student acquaintance with taking the prior unit), and teacher experience (previous teacher acquaintance with teaching the curriculum) displayed nonsignificant *P*-values in all but one or two instances.

However, class hour, recorded for each class session, attained statistical significance in six instances of regression output pertaining to three of the four 2017–18 semesters. For the three teachers who covered the *HiOH* curriculum for more than 2 class hours during a semester, class hour was a statistically significant variable for student survey question items 1 (importance to everday life) and 5 (information obtained outside of the class is useful).

## Discussion

### Broadening student knowledge

Prior middle and high school educational studies teaching biology both in and outside the classroom have identified learning of particular scientific concepts or skills, such as the generation of traits and characteristics (phenotypes) from genes and proteins and using evidence to support claims, and general life skills such as working with others as important benefits perceived by students (Alozie et al. 2010; Luehmann 2009). Our student and teacher surveys registered gains for the two curricular units in interest in the environment, perceived usefulness of what was learned, with learning about health and health conditions/disease showing the highest gains in both units, and importance of genetic knowledge to everyday life. Scientific practices such as analyzing and interpreting data, constructing explanations, and developing and using models, particularly as they applied to the effect of genes and the environment on health, were mainstays of the curriculum. For the diabetes unit, the “environment” referred to diet and nutritional intake, and to opportunities for physical exercise. For the SUD unit, environment referred to exposure to various sources of addiction, including the social media milieu. Improvements in teacher survey ratings through time suggest that teachers were increasingly satisfied with the program’s educational value as they gained familiarity and teaching experience with it.

### Subgroup analysis insights

The regression analysis showed that class hour was a statistically significant variable in pre- /post-differences in student responses to survey questions dealing with importance to everyday life and usefulness of outside information. Whether this differential was due to one class being more talkative and engaged in information sharing than another or intentional set-up of the classes bears further inquiry—time of day (a teacher working out kinks in lesson delivery with each successive same-day class) displayed no consistent pattern.

Sex was a statistically significant variable in only two instances in the student surveys we administered (*P* < 0.005 - see Table 3; *P* < 0.05 - Table 4). Male students displayed a greater pre-post gain than female students (difference of 17.8%) in the frequency of those strongly agreeing that science class is useful for learning about health and disease during the fall 2018 substance use enactment. However, females displayed a greater pre-post gain for this statement during the spring enactment. Also, more male students displayed a greater pre-post gain than female students in the frequency of those strongly agreeing they learned how genetic knowledge can be important to everyday life during the fall 2017 diabetes enactment, but the difference between sexes in the frequency of those displaying such a gain was only 4.8%. If anything, female students were much more likely during the diabetes curriculum pilot phase student interviews than their male counterparts to communicate their awareness of the existence of diabetes in the family and steps taken by family members. At the grade levels we assessed, a male–female skew was not apparent in perceived learning for either unit.

STEM literature depicts a predominance of males earning science degrees and entering the technical job market (Kuchynka et al. 2022; Kong et al. 2020). The even breakdown in student representation by sex within our classrooms helped assure equal exposure of both males and females to a science curriculum. Proposals to increase gender-unbiased student interest in STEM fields that we have used in our own project include the use of diverse role models, science-themed extracurricular programs, empowering female students to get involved in “real-world” issues and situations, and emphasis on communal goals (Kuchynka et al. 2022; Kong et al. 2020). The addition of arts to the STEM curriculum has been shown to increase appeal (Wajngurt and Sloan 2019). We encouraged students to show their learning in a variety of ways, including poster design and rap or hip-hop during presentation events.

### Student ability to identify with phenomena of varied complexity

In general, the proportion of students strongly agreeing that they learned from the curriculum was 10–20% higher for the diabetes unit, which was developed earlier than the substance use disorder unit and had gone through more revisions during its three-semester implementation. Additionally, the SUD material, which covered the neurological-behavioral domain, was more complex overall than the diabetes material (MacNabb et al. 2006). Transitioning between different levels of biological organization—cell, tissue, organ, and organism—can be a challenge. Additionally, complex traits such as behavioral responses, which are not well addressed in state educational standards (in higher education, the requisite concepts of continuous variation, polygenic inheritance, and multifactorial causation are dealt with), though a grasp of environmental factors (physical and lifestyle-related) interacting with genetic information is an essential part of the learning progression in modern genetics and is crucial from a public health perspective (Dougherty et al. 2011; Duncan et al. 2009).

### Student and adult interest in in-class investigations and community research projects

As depicted in the current findings and those of the prior SEPA iteration with high school students that included DNA transcription and translation, understanding of in-class material is influenced by the complexity of the phenomena discussed and student ability to personally identify with the models used (Alozie et al. 2010; Duncan et al. 2009). Students frequently mentioned the example of Monique, an individual their own age, in discussions with parents, but also showed a great deal of pride in the models they constructed to explain the phenomena of diabetes or SUD and subsequently used at the culminating Health Summit events.

Students liked the community action research projects, especially smoothie tasting. In informal discussions at the presentation events, students also enjoyed the chance to hear about other students’ projects, including from other schools, and to respond to judges’ questions about their own projects. In the SUD presentation event questionnaires, students voiced appreciation of each others’ help in presenting models of the brain’s reward system. This finding agrees with an earlier Minnesota program that taught neuroscience in middle school in which students voiced a desire to interact more with one another on brain activities (MacNabb et al. 2006) Such enthusiasm for in-class projects tended to be a part of student comments but appeared less frequently in the parent interviews and questionnaires than mention of the CAPs. Nevertheless, tallies showed room exists for more parental involvement in the CAPs. Questionnaire results indicated that standard e-mail is the most effective way to initially apprise parents of what is happening in science class.

### Lessons learned

The diabetes unit pilot study highlighted the need to motivate students to share what they were learning with friends and family. Following the 2016 pilot, we approached this challenge in 3 ways: (1) the curriculum was supplemented by “connection to student’s lives” and “family engagement” sections that included examples of discussions and activities that could be done at home; (2) the importance of such interactions was reinforced in teacher professional learning by discussing these sections and getting teacher feedback; and (3) community partners encouraged discussions on the varied places and ways to engage families outside the classroom setting. These actions contributed to the improvements noted in 2017 and 2018. Student and teacher field testing surveys and interviews with parents showed that what the students learned was brought home and shared with family, and that in some instances, the student’s knowledge and enthusiasm translated into a greater health focus at home. The findings on student sharing reflect the value of a multipronged approach to introduce curricular improvements in collaboration with teacher and community partners.

Following the first round of SUD unit pilot testing, a career panel was organized where adult volunteers explicitly discussed their STEM occupations and relevance to the day’s events and what the students were learning. This move represents a shift away from our earlier use of a physician as the sole career model (biology learning center programs have used scientists for this purpose) to a more community-embedded (e.g., a county health educator, health plan community liaison, and nearby health research center program manager) set of career models (Alcena-Stiner and Markowitz 2020; Cold Spring Harbor Laboratory 2020). The use of community mentors and career panelists overcomes the learning obstacle noted by Bouillion and Gomez that “Schools are in communities but often not of communities” (Bouillion and Gomez 2001). As evinced by the fall 2018 event tabulations and open-ended responses, this format aided participant appreciation of future job and educational possibilities.

Teacher and administrator turnover were responsible for the loss of personal relationships that had supported our engagement with one of the Detroit schools. Podolsky et al. (2019), using National Center for Education Statistics, show that among 14 categories of reasons, 17% of US teachers who leave their positions credit lack of administrative support to address disciplinary problems for their decision; 14% attribute lack of autonomy in organizing and conducting lessons; and 13% state insufficient salary as a reason. The educational literature further suggests professional development (PD), providing familiarizing coursework for new STEM teachers and those teaching new STEM material, can be a facilitator of teacher retention (Hayes et al. 2019; Podolsky et al. 2019; Allen and Sims 2017). Lack of resources (insufficient teaching supports and understaffing), especially in under-resourced schools, can hinder such efforts, though the literature is mixed regarding its impact on retention versus teaching morale (Podolsky et al. 2019; Buckley et al. 2004). In our program, two to 3 staff members per year provided PD to teachers to maximize its effectiveness. These individuals made classroom visits and were also available by e-mail and cell phone for on-the-spot support. Our Professional Learning Communities sessions initially entailed 3 days of face-to-face professional learning, advanced to weekly in-person and online meetings, and were followed up later in the term. Feedback from teachers and administrators indicated this level of support generally satisfied their needs and the personalized access to support was especially effective and valued.

A qualitative study by Kokka of an under-resourced urban school identifies social emotional rewards from student interactions as a factor in the longevity of a teacher’s stay (Kokka 2016). Our efforts duplicated this finding—the biggest teacher incentive was its visible impact on student learning. Teachers became committed when they experienced firsthand that their students were motivated and engaged in science learning. The teachers felt their efforts validated in seeing the critical thinking that took place in the community action research projects and at the Health Summits.

### Communication with students’ families

In a study of 22 middle and high schools partnering the districts’ student information systems and teacher gradebooks with parent text messaging when students are having academic difficulty, educational researchers found student course failures decreased by 38% and class attendance increased 17% (Bergman and Chan 2017). The study concluded, “Informed parents can play an important role in increasing student achievement for those struggling more in school” (p. 21). Our program uses Facebook, Instagram, and Twitter, as well as traditional means to inform parents about curriculum happenings. Pew Research Center studies show that 75% of parents use social media with Facebook and Instagram as the top two sources, compared to 95% of teens using social media, YouTube and TikTok being their top two sites (Vogels et al. 2022; Auxier and Anderson 2021; Duggan et al. 2015).

A paradoxical finding in a mixed-methods study of technology use in California schools is that while 62.1% of parents indicated they would be a “friend” on a school Facebook page and 54% that they had used e-mail to communicate with teachers, only 24.1% were interested in “following” the school on the social media site Twitter (Olmstead 2013). Studies reporting decreased social media use in adults of lower income and parental preference for e-mail and phone use for communications between home and school (Hruska and Maresova 2020; Olmstead 2013; Zieger and Tan 2012) provide a partial explanation of our finding that parents were less receptive to social media messaging than are young social media active students (Vogels et al. 2022).

### Limitations

Project involvement in Detroit did not proceed beyond pilot and early field testing. In Flint and Detroit, although those attending the presentation events were involved in their student’s projects, only a fraction of parents were present at these events, suggesting more needs to be done with outreach than moving the event to evenings and supplying meals. These two limitations hinder generalizability. The analysis failed to show that teacher experience in terms of prior semesters taught and student experience with respect to contact with both units were significant contributors to the outcomes.

### Future studies

Based on our findings regarding means of notifying families, we suggest that educators emphasize e-mail and traditional modalities (e.g., phone, including text messaging) as primary means to inform parents about curriculum happenings, with social media as an adjunct. Project coordinators can spread news of the curriculum at events and family meetings at the school and in the community. Nevertheless, students and their families at this point use social media. Especially during the COVID-19 pandemic, it was important to be able to connect the curriculum with students online and to organize the Health Summit presentation events online/virtually. Students can find presenting online challenging; these avenues are responsive to circumstances but are not meant to supplant face-to-face engagements. We intend to selectively use both in-person and online modalities for our students in future iterations.

In terms of research outcome, future studies might be extended an additional year to increase the N for experience-related covariates. Value also exists in extending tracking of student science class performance beyond the 8th grade to more fully assess intermediate-term impact. Finally, in addition to self-reported gains in interest around the phenomena and perceived usefulness of the lessons, measuring students’ objective knowledge gains would be of complementary value to the educational community. Future studies are looking to assess students’ pre and posttest scientific knowledge.

## Conclusion

Health in Our Hands focused on public health phenomena and utilized multiple educational approaches—classroom discussions, in-class investigations, simulations and modeling activities, and community action research projects—to teach the interaction of genes and the environment within middle school science classes in underserved communities. Strengths of the program were that it (1) demonstrated increases in positive student responses for several measured categories—value of science for everyday life, perceived learning, and interpersonal sharing; (2) engaged students in different conditions in varied educational settings—formal classroom and informal community-connected; and (3) showed success in medically underserved counties with elevated risk for the conditions under focus (Michigan Department of Health and Human Services 2021; Michigan Department of Community Health, Mental Health and Substance Abuse Administration 2010).

Importantly, Health in Our Hands demonstrates an effective model for applying modern genetic concepts to critical public health issues in school settings. The curriculum content addressed the Michigan Science Standards (MSS) for grades 6–8 with respect to environmental and genetic factors and interacting systems, selective adaptation, and brain messaging (Michigan Department of Education 2015). It also impacted student awareness of educational and career possibilities in science. In addition to science learning, the program touched areas covered in middle school health education. The 6th and 7th–8th grade content expectations in Michigan include healthy eating and physical activity strategies, understanding the influences on and consequences of tobacco, alcohol, and other drug use, analyzing the influence of computer use on physical activity (7th–8th grades), strategies to support youth who have diabetes (7th–8th grades), and advocacy skills for personal, family, and community health (Michigan Department of Education 2006). Healthy eating and physical activity practices and programs that partner with families and community members are also propounded by the CDC School Health Guidelines (NCCDPHP 2011). These content items, part of the Health in Our Hands curriculum, are relevant to the age group assessed and communities chosen. That is, learning was assisted through a focus on phenomena that occur in the lives of students, their families, and communities, constituting what Bouillion and Gomez have called “connected science” (2001). Genetic literacy itself involves responding to information about genetic phenomena that one may encounter in everyday life situations (Duncan et al. 2009). To effectively extend education beyond the classroom, further concerted efforts are needed to engage family members. Longitudinal analysis, which is the current evolution of the Health in Our Hands effort, can be useful for exploring broader project impact.

The project was subtitled “A New Genomic Framework for Schools and Communities.” Activities combined detailed curriculum, teacher professional learning, and community partnership to support and sustain its various segments. Teaching diabetes personal and family history and the neurophysiological/adaptive basis of addictive behavior are new themes for the middle school years. As an educational tool, computer simulations can be a useful adjunct to promote inquiry in this academic area and school setting (Damelin et al. 2017; Dou 2017). Student interest can vary—inclusion of a variety of project types and means for students to present their projects (e.g., through graphics, photos, and rap) is advisable (Kuchynka et al. 2022; Shim and Lee 2019). Given that substance use constituted a major portion of our curriculum, it is important to hold in mind that teen substance use prevention programs have been found to be most successful when both peers and adults are involved (Nation et al. 2003). Community mentor-student partnering was a critical enabler of projects covering these areas in the informal educational setting.

Authors have described a general decline in the quality of the parent-adolescent relationship between middle and high school, with the norm being an increasing sense of alienation and decreasing trust (Ebbert et al. 2019; Steinberg and Morris 2001). We feel that in our program, family involvement during the 6th through 8th grade years has occurred at an opportune time in child development when students remain open to their parents’ participation. To continue to expand education beyond the classroom, further concerted efforts are needed to engage family members. While findings did indicate that students communicated more with parents following the units, raw percentages showed that extra efforts must be taken to promote sharing. Likewise, the means used to communicate with parents are important; students respond to messaging through different avenues than adults. Adoption of innovative educational frameworks enables relevant community health issues, such as diabetes and substance use disorder, to serve as useful biological phenomena for anchoring middle school genetics education and discussion connecting students, family, and community members.

## Data Availability

No additional data is available beyond the article contents.
